# Differential endothelial cell cycle status in postnatal retinal vessels revealed using a novel PIP-FUCCI reporter and zonation analysis

**DOI:** 10.1007/s10456-024-09920-0

**Published:** 2024-05-25

**Authors:** Ziqing Liu, Natalie T. Tanke, Alexandra Neal, Tianji Yu, Tershona Branch, Arya Sharma, Jean G. Cook, Victoria L. Bautch

**Affiliations:** 1 Department of Biology, CB 3280, The University of North Carolina at Chapel Hill, Chapel Hill, NC 27599 USA; 2 Curriculum in Cell Biology and Physiology, The University of North Carolina, Chapel Hill, NC USA; 3 Department of Biochemistry and Biophysics, The University of North Carolina, Chapel Hill, NC USA; 4 McAllister Heart Institute, The University of North Carolina, Chapel Hill, NC USA; 5 Lineberger Comprehensive Cancer Center, The University of North Carolina, Chapel Hill, NC USA; 6 Present Address: Department of Physiology, Medical College of Wisconsin, Milwaukee, WI 53226 USA

**Keywords:** Cell cycle, Endothelial cells, HUVEC, Blood vessel, G2 arrest

## Abstract

Cell cycle regulation is critical to blood vessel formation and function, but how the endothelial cell cycle integrates with vascular regulation is not well-understood, and available dynamic cell cycle reporters do not precisely distinguish all cell cycle stage transitions in vivo. Here we characterized a recently developed improved cell cycle reporter (PIP-FUCCI) that precisely delineates S phase and the S/G2 transition. Live image analysis of primary endothelial cells revealed predicted temporal changes and well-defined stage transitions. A new inducible mouse cell cycle reporter allele was selectively expressed in postnatal retinal endothelial cells upon Cre-mediated activation and predicted endothelial cell cycle status. We developed a semi-automated zonation program to define endothelial cell cycle status in spatially defined and developmentally distinct retinal areas and found predicted cell cycle stage differences in arteries, veins, and remodeled and angiogenic capillaries. Surprisingly, the predicted dearth of S-phase proliferative tip cells relative to stalk cells at the vascular front was accompanied by an unexpected enrichment for endothelial tip and stalk cells in G2, suggesting G2 stalling as a contribution to tip-cell arrest and dynamics at the front. Thus, this improved reporter precisely defines endothelial cell cycle status in vivo and reveals novel G2 regulation that may contribute to unique aspects of blood vessel network expansion.

**Supplementary Information:**

The online version contains supplementary material available at 10.1007/s10456-024-09920-0.

## Introduction

Heterogeneity of endothelial cell responses to signaling inputs is crucial for sprouting angiogenesis and blood vessel network expansion [1–3]. For example, differential Notch signaling is important for defining tip cells vs. stalk cells in emerging sprouts [4], and Notch status also determines the response of endothelial cells to pro-angiogenic BMP signals [5]. The cell cycle is temporally regulated in actively cycling cells through G1-S-G2-M stages [3, 6, 7] and recent work shows that venous identity is linked to cell cycle gene regulation [8, 9], venous/lymphatic sprouting is regulated in G1 [10], and that cues for arterial vs venous sub-type differentiation are differentially processed in early G1 vs. late G1 [11, 12]. Cells often experience a temporary cell cycle arrest (called G0 or extended G1) and are considered quiescent [11, 13, 14], and endothelial tip cells are thought to be in a temporary cell cycle arrest due to high VEGF-A signaling [15]. Although vascular programs link to the cell cycle, how cell cycle status contributes to endothelial cell heterogeneity in signaling responses and vessel network expansion is poorly understood.

The dynamic nature of cell cycle transit poses challenges for precise staging. Investigators have used short sequences encoding information in cis, called degrons, that mediate protein degradation during the cell cycle [16, 17]. When degrons are linked to fluorescent reporters, time-dependent cell cycle changes are documented with spatially relevant readouts. The original FUCCI reporter distinguishes G1/G0 from S/G2/M and has been incorporated into cells and animals such as flies, fish, and mice [18–20]. Original FUCCI mice exhibited variable reporter expression due to random transgenesis, so R26R-FUCCI2 mice were generated using mCherry-hCdt1_30-120_ and mVenus-Geminin_1-110,_ which expressed FUCCI reporters bidirectionally from the ROSA26 locus [21]. The R26-FUCCI2aR mouse ensured conditional expression of both FUCCI reporters in the same ratio [19, 22]. Newer FUCCI reporter versions utilized Cdt1_1-100_ that included the PIP degron and improved some transitions but contained other potential binding regions that may interfere with endogenous cell cycle, and reporter mice carrying these versions were not reported [23]. A more recent version utilized here has mCherry-Gem_1-110_ expression tightly confined to S and G2, along with a smaller fragment of Cdt1, Cdt_1-17_, that contains the PIP degron and whose overexpression is not predicted to affect the endogenous cell cycle; moreover, this fragment expresses precisely at the start of G2, with degradation precisely at the start of S phase [24]. This improved reporter, called PIP-FUCCI, distinguishes S, G2, and G1/G0, and it has been extensively validated in cultured transformed cells and found to reflect cell cycle status.

Here we investigated this cell cycle reporter in primary endothelial cells and in developing murine blood vessels in vivo. We found concordance with established cell cycle readouts and for the first time precisely define S and G2 phases in endothelial cells in vivo. A novel semi-automated zonation pipeline revealed that different spatial domains of the expanding retinal vascular plexus had different cell cycle stage distributions, and we documented an unexpected enrichment of endothelial tip and stalk cells in G2, suggesting how the endothelial cell cycle may integrate with vascular morphogenesis.

## Materials and methods

### Endothelial cells and imaging

HUVEC (Lonza #C2519A) were cultured according to the manufacturer’s recommendations in EBM2 (CC-3162, Lonza) supplemented with added growth factors (Bullet kit, CC-3162, Lonza, referred to as EGM2) at 37 °C/5% CO_2_, and infected with PIP-FUCCI (Addgene, #118621) or H2B-CFP lentivirus at ≤ P (passage) 4. HUVEC were incubated with 1 mL of viral supernatant (prepared as described [24] in media containing 8 µg/mL Polybrene (Sigma, TR-1003-G) for 4 h, media was replaced for 24 h, then HUVEC were seeded onto glass-bottom plates for live imaging.

Images were acquired as previously described [24]. Briefly, cells were housed in a humidified chamber (Okolabs) at 37 °C/5% CO_2_ for 48 h with image acquisition at 10 min intervals using a Nikon Ti Eclipse inverted microscope and 20 × objective lens (NA 0.75). No photobleaching or phototoxicity was observed using this protocol. Images were processed and tracked in ImageJ. Endothelial cells in the imaging field for one or more phases were chosen for phase measurements, and cells that remained in the imaging field through an entire cell cycle (mitosis to mitosis, scored by co-expression of H2B-CFP) were used for total cell cycle tracks.

### Mice and breeding

**Fig. 1:**
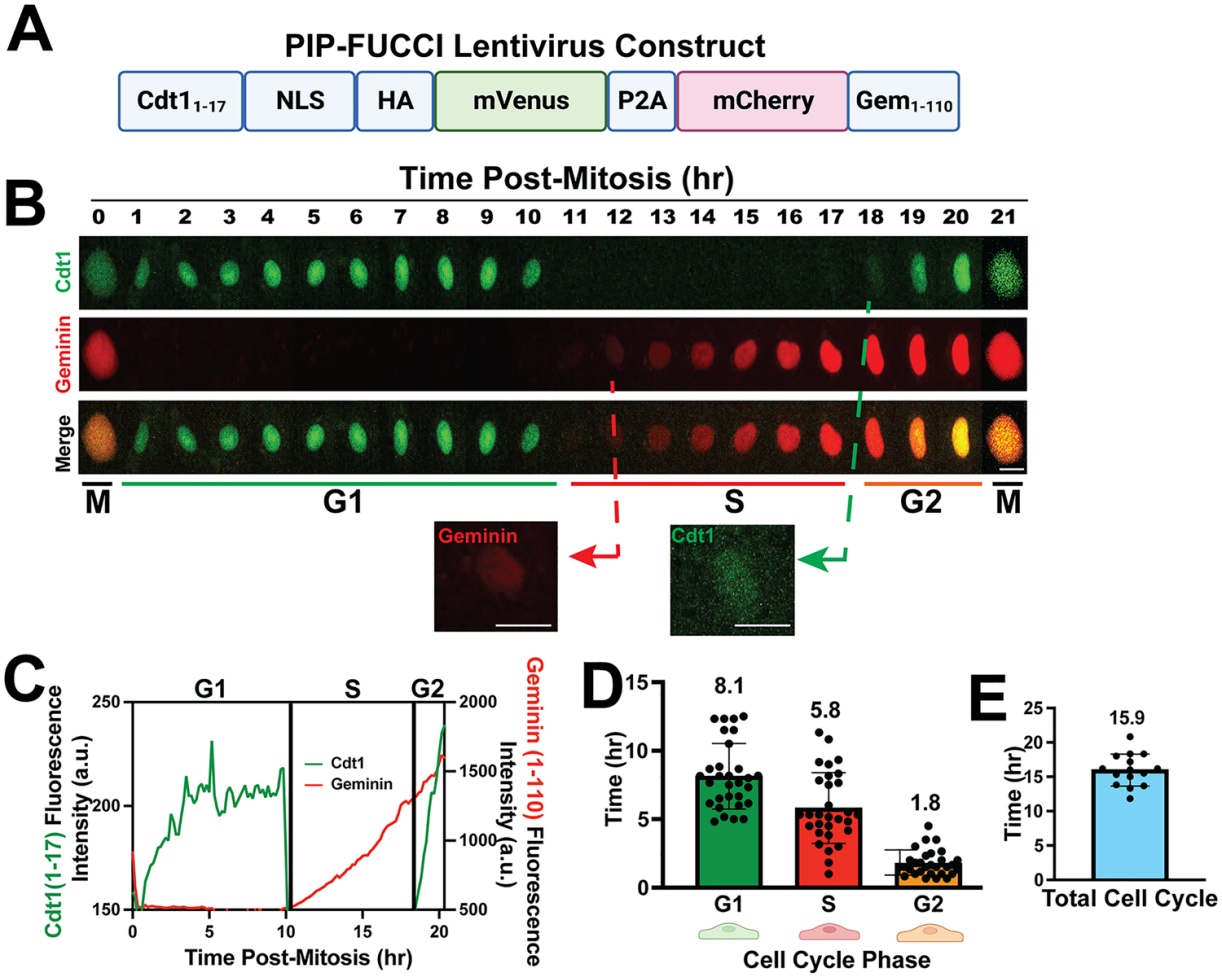
PIP-FUCCI Lentivirus reports cell cycle status in HUVEC. **A** PIP-FUCCI lentivirus construct. NLS, nuclear localization signal; HA, HA tag; P2A, self-cleaving peptide P2A; Gem, geminin. **B** Representative time-lapse images of PIP-FUCCI transduced HUVEC, showing mVenus-Cdt1_1-17_ (green) and mCherry-Geminin_1-110_ (red) expression hourly from end of cytokinesis (M) through next cytokinesis. Scale bar, 20 μm. **C** Quantification of PIP-FUCCI fluorescence intensity/time from cell in **B**. **D** Average time spent in each cell cycle phase (hr). (n = 30 cells per phase from 3 replicate movies) **E**. Total endothelial cell cycle length, time between mitoses measured by H2B-CFP. (n = 15 cells from 3 replicate movies)

All animal experiments were approved by the U. North Carolina at Chapel Hill (UNC-CH) Institutional Animal Care and Use Committee. Mice were generated and maintained on the C57BL/6J genetic background, and pups of both sexes were included in the analysis. *Cdh5-Cre*^*ERT2*^* (Tg(Cdh5-cre/ERT2)1Rha*) mice [25] were obtained from Cancer Research UK. The new PIP-FUCCI knock-in reporter line (*C57BL/6J-Gt(ROSA)26Sor*^*em1(CAG-LSL-PIP-FUCCI)Vb*^*/Vb,* called PIP-FUCCI (PF)) was generated by the UNC-CH Animal Models Core via CRISPR/Cas9-mediated genome editing as described [26] with modifications. Briefly, the PIP-FUCCI DNA (Fig. 1A) was amplified via PCR, and the amplicon was cloned into a Rosa26 gene targeting construct customized for targeting with the guide RNA (Supp. Fig. 1D). The targeting construct contained a splice-acceptor/neomycin resistance cassette, CAG promoter, LoxP-STOP-LoxP cassette (with puromycin resistance gene), PIP-FUCCI coding sequence, Woodchuck Hepatitis Virus posttranscriptional regulatory element (WPRE), and rabbit β-globin polyadenylation sequence. The construct, guide RNA and Cas9 protein mixture was injected into C57Bl6/J zygotes that were then implanted into pseudo-pregnant females. Positive founders were bred to heterozygosity or homozygosity for subsequent experiments. Mice carrying the PIP-FUCCI allele (*PF/PF* or *PF/*+) were bred to *Cdh5-Cre*^*ERT2*^ mice.

To induce genetic deletion, 50 µl of 1 mg/ml tamoxifen (Sigma T5648, 30 mg/kg body weight) dissolved in sunflower oil was injected intraperitoneally (IP) into *PF/PF;Cdh5Cre*^*ERT2/*+^ or *PF/*+*;Cdh5Cre*^*ERT2/*+^ pups on P (postnatal day)1-P3 [27]. Genotyping primers amplified either wildtype *Rosa26* locus (WT-F & WT-R) or locus with PIP-FUCCI insert (PF-F & PF-R, Supp. Fig. 1E–F, Supp. Table 1).

### Retinal staining and EdU labeling

Tamoxifen-injected pups were sacrificed at P6, eyes were collected, fixed in 4% PFA for 1 h at RT (room temperature), then dissected and stored at 4 °C in PBS. Staining and imaging were performed within one week of sample collection to avoid fluorescence quenching. Retinas were permeabilized in 0.5% Triton X-100 (T8787, Sigma) for 10 min at RT, blocked for 1 h at RT in blocking solution (5% rabbit serum (10510, ThermoFisher) and 1% BSA (A4503, Sigma) in TBST (0.1% Tween-20 in 150 mM NaCl, 50 mM Tris–HCl, pH 7.4)), then incubated with IB4 (Isolectin B4)-biotin in blocking solution overnight at 4 °C. Samples were washed 3X with TBST, then incubated with Streptavidin-Alexa405 and/or ERG-Alexa647 or Ki67-Alexa647 antibody for 1 h at RT (Supp. Table 2). Retinas were mounted with Prolong Diamond Antifade mounting medium (P36961, Life Technologies) and sealed with nail polish.

For EdU labeling, 50 µl of 3 mg/ml EdU (Thermo Fisher, A10044, 50 mg/kg body weight) dissolved in PBS was injected IP into P6 pups 2 h prior to harvest [28, 29]. EdU staining was performed with the Edu-Click-iT-Alexa647 kit (C10340, Thermo Fisher) prior to staining with additional antibodies. Briefly, after the permeabilization step above, retinas were washed 2 × with 3% BSA/PBS and stained with the Click-iT reaction solution at RT for 30 min. Standard antibody staining for IB4 or ERG was then performed prior to mounting.

### Retina image analysis

Confocal images were acquired with an Olympus confocal laser scanning microscope and camera (Fluoview FV3000, IX83) using 405, 488, 561, and 640 nm lasers and 20 × objective. The entire retina was imaged by collecting 5 × 5 or 6 × 6 fields and stitching. Image analysis was performed manually for EdU labeling and Ki67 staining by counting PIP-FUCCI-labeled endothelial cells from 5 to 8 20 × images per pup. Image analysis for PIP-FUCCI/ERG co-stain followed a novel semi-automated pipeline with custom scripts in Fiji and R [30, 31]. GitHub (https://github.com/BautchLab/Liu-2024-Angiogenesis) has custom Fiji and R scripts and a detailed retinal zonation protocol. The workflow of PIP-FUCCI/ERG whole retina image analysis (Supp. Fig. 1G–H) and vascular zonation analysis (Supp. Fig. 2D) was as follows. Briefly, stitched images were processed in image J to obtain mean fluorescence intensity (MFI) of mVenus and mCherry in each endothelial cell nucleus, using ERG stain as a mask. Each nucleus was assigned a specific ID and the x–y coordinates recorded. Next, vascular zones were manually drawn as polygons for each retina based on the following criteria: primary arteries (PA) and veins (PV) started from the optic disk and ended at the first bifurcation; arterioles (Art) and venules (Ven) branched out from PA and PV and connected to a capillary bed with uniform vessel diameter; tip cells (Tip) were sprouting endothelial cells at a “tip” position located in the very first row of the angiogenic front; stalk cells (Stalk) were non-sprouting endothelial cells either immediately behind or adjacent to a tip cell; angiogenic front capillaries (AFC) were the 3–5 rows of endothelial cells of the angiogenic front behind tip cells and did not include stalk cells; mature capillaries (MC) were capillaries behind the AFC. The x–y coordinates of each vascular zone polygon were recorded except for tip and stalk cells, whose nucleus ID was manually recorded. The MFI and x–y coordinates for each endothelial nucleus, and the x–y coordinates for each vascular zone were imported into R for whole retina or zonation analysis. For quantification, data from all polygons of each vascular zone were merged for each retina, and then data from each retina was plotted. The classification of vascular zones is mutually exclusive for each endothelial nucleus. All PIP-FUCCI labeled and ERG+ nuclei were included for quantification using our pipeline. For zonation analysis, 2/4 retinas had no tip cells in S and 11% and 13% of tip cells scored in G2. Because indefinite numbers cannot be input for calculations and statistics, we set the G2/S ratio of these retinas in tip category at 10 (ratio was 9 and 8 for the other 2 retinas).

## Results and discussion

A new PIP-FUCCI construct that contained only the first 17 amino acids of Cdt1 linked to mVenus, along with amino acids 1–110 of Geminin linked to mCherry, was shown to precisely distinguish G1/S and S/G2 in U2OS cells [24], and here we examined primary endothelial cells expressing the reporter via lentivirus infection (Fig. 1A). Live image analysis revealed sharp transitions for Cdt1_1-17_mVenus, with degradation at G1/S and re-expression at S/G2 (Fig. 1B–C, Supp. Movie 1), and mitosis always followed G2 in cells imaged to this stage transition. Analysis of multiple endothelial cells revealed that G1 phase averaged 8.1 h, S phase averaged 5.8 h, and G2 averaged 1.8 h, adding to a total endothelial cell cycle average of 15.7 h (Fig. 1D), in good agreement with total cell cycle transit of 15.9 h, as scored by time between mitoses of H2B-CFP expressing HUVEC (Fig. 1E). As described in Grant et al. [24], the degradation of Cdt1_1-17_mVenus at G1/S was considered an exact measure of the start of S phase (as defined by formation of PCNA foci), and different cell lines sometimes showed a lag of Gem_1-110_ accumulation; a slight lag was documented in HUVEC, likely due to signal accumulation for Gem_1-110_. The G2/S ratio was 0.3, consistent with G2 being shorter than S in the cell cycle. Thus, primary endothelial cells regulate the PIP-FUCCI reporter in a temporal manner consistent with their cell cycle transit.

We next tracked labeled primary endothelial cells over time and asked whether cell cycle stage affected migration dynamics. We found that endothelial cells in G1 showed increased velocity and migrated distance/time (normalized to the average G2 interval) compared to cells in S-phase or G2 (Supp. Fig. 1A–C; Supp. Movies 2–4). This finding is consistent with another study showing enhanced G1 migration in cancer cells [32] and suggests that once G1 is complete, endothelial cell migration slows but does not completely stop under culture conditions, perhaps to accommodate activities supporting DNA synthesis and preparation for mitosis.

**Fig. 2:**
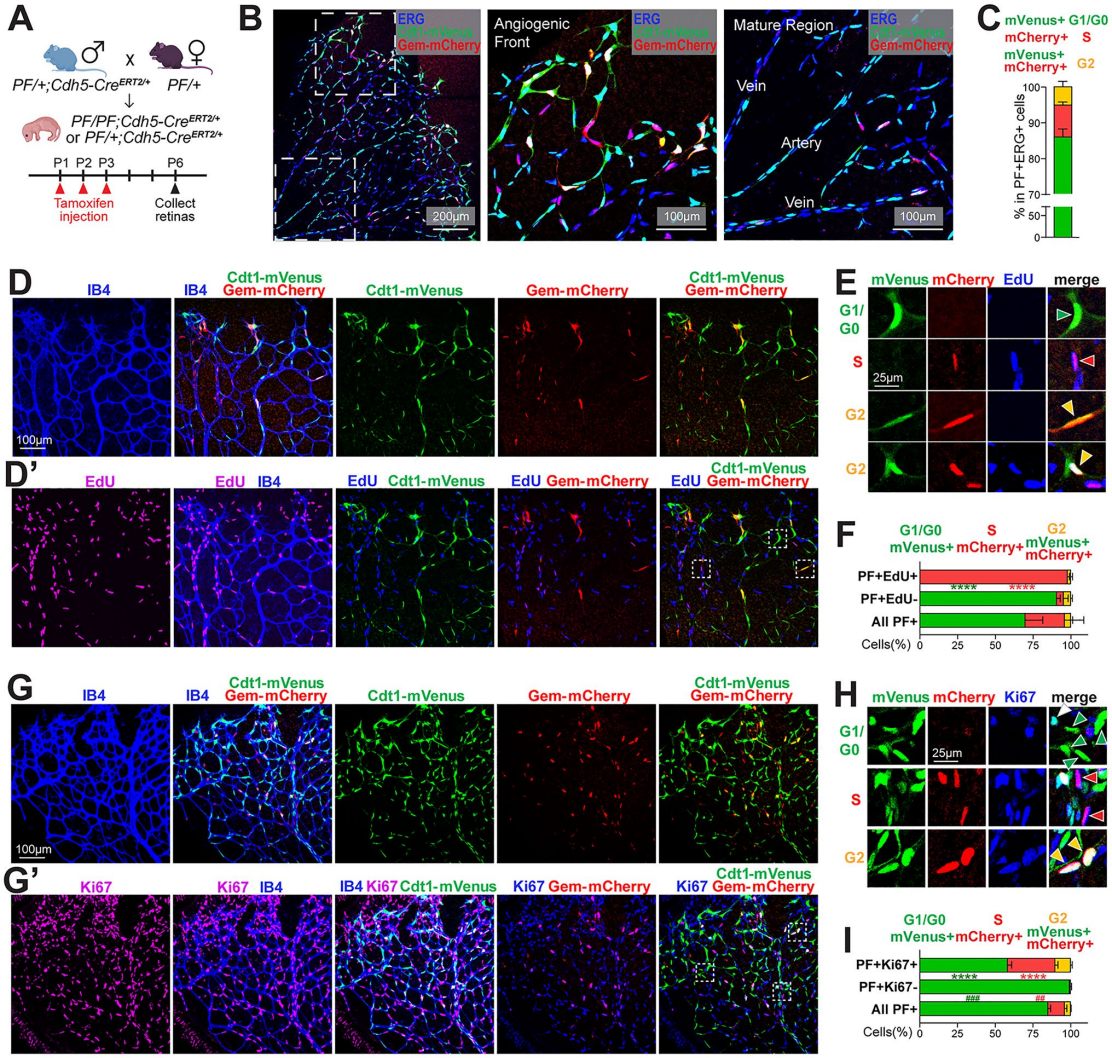
PIP-FUCCI mouse reports cell cycle status in postnatal retinal vessels in vivo*.* **A** Breeding scheme and schedule for *PF/PF;Cdh5-Cre*^*ERT2/*+^ and *PF/*+*;Cdh5-Cre*^*ERT2/*+^ pups. **B** Representative images of one leaflet of a *PF/PF*;*Cdh5-Cre*^*ERT2/*+^ retina stained for ERG. Boxed areas in far left panel (scale bar, 200 μm) are magnified (scale bar, 100 μm) in middle (Angiogenic Front) and far right (Mature Region) panels. **C** Quantification of whole retina endothelial cell cycle phase analysis from PIP-FUCCI labeled P6 retinas stained for ERG (blue). n = 4 pups. **D–F** Representative images (**D–E**) and quantification (**F**) of P6 PIP-FUCCI labeled retinas stained for IB4 (blue) and EdU-labeled (purple or blue). **E** Representative cells (arrowheads) PIP-FUCCI labeled as in G1/G0 (green), S (red), or G2 (yellow) cell cycle phase, magnified from white boxed areas in **D’** (top 3 rows of E) and another area of the same retina (bottom row). Scale bar (**D**) 100 μm; (**E**) 25  μm. **F** Indicated quantification, n = 3 pups. ****p < 0.0001 by Two-way ANOVA & Sidak’s multiple comparisons test comparing PF+EdU+ and all PF+ cells. **G–I** Representative images (**G–H**) and quantification (**I**) of PIP-FUCCI labeled P6 retinas stained for IB4 (blue) and Ki67 (purple). **H** Representative cells (arrowheads) PIP-FUCCI labeled as in G1/G0 (green), S (red), or G2 (yellow) cell cycle phase, magnified from white boxed areas in **G’**. Scale bar (G) 100 μm, (H) 25 μm. **I** Indicated quantification, n = 2 pups. ****p < 0.0001 by Two-way ANOVA & Sidak’s multiple comparisons test comparing PF+Ki67+ and all PF+ cells; ###p < 0.001, ##p < 0.01 comparing PF+Ki67- and All PF+ cells

To generate a mouse carrying an inducible PIP-FUCCI allele, the PIP-FUCCI construct was placed 3’ to a standard cassette and built into the ROSA26 locus via CRISPR/Cas9-mediated insertion (Supp. Fig. 1D, see Methods). Mice that were either heterozygous or homozygous for the allele *C57BL/6J-Gt(ROSA)26Sor*^*em1(CAG-LSL-PIP-FUCCI)Vb*^*/Vb*, *hereafter* called PIP-FUCCI (*PF/*+ or *PF/PF*) were bred to *Tg(Cdh5-cre/ERT2)1Rha* (hereafter referred to as *Cdh5-Cre*^*ERT2*^) mice to excise the lox-STOP-lox cassette and induce reporter expression in endothelial cells of early post-natal retinas (Fig. 2A, Supp. Fig. 1E–F). Overlay of the PIP-FUCCI reporter signal with Isolectin B4 (IB4, vascular-specific, Supp. Fig. 2C–C’) or ERG (vascular endothelial-specific, Fig. 2B) staining of P6 retinal vessels showed that the PIP-FUCCI signal labeled only IB4- or ERG-positive cells, indicating endothelial cell-specific expression of the reporter in the vasculature in vivo.

To quantitatively analyze endothelial cell cycle in the postnatal retina with PIP-FUCCI, we created an ERG mask for all retinal endothelial cells (Supp. Fig. 1G) that allowed for determination of mVenus and mCherry nuclear intensity in each individual endothelial cell. We then developed a semi-automated image analysis protocol (Supp. Fig. 1H) that assigned each endothelial cell to a cell cycle phase (G1/G0, S, or G2) based on PIP-FUCCI reporter fluorescence. This analysis showed that most endothelial cells (82% for *PF/PF* homozygous retinas and 62% for *PF/*+ heterozygous retinas, Supp. Fig. 1I) were labeled, with the small proportion of unlabeled cells likely a combination of unexcised lox-STOP-lox cassette and a few cells in very early G1 or right at the G1/S transition. Unlabeled endothelial cells were not assigned a cell cycle status or used in the quantification. Manual validation of cell cycle phase assignment using a subset of endothelial cells showed that our pipeline was accurate 96% of time (Supp. Fig. 1J). Using this pipeline, we identified the overall proportion of endothelial cells in G1/G0 vs. S vs. G2 in the retina to be 86:9:5 (Fig. 2C), consistent with previous reports [11]. G1/G0 is longer than S and G2, as we found in cultured HUVEC (Fig. 1), although the retinal analysis revealed a lower ratio of G2 (0.06) or S (0.1) to G1/G0 cells, likely due to numerous endothelial cells that were in extended G1/G0 (quiescence) in vivo but not found in actively cycling cultured endothelial cells.

We next examined the relationship of the PIP-FUCCI reporter with EdU-labeling, which identifies cells in S-phase during the labeling period (Fig. 2D–F, Supp. Fig. 2A). Among PIP-FUCCI labeled retinal cells, mVenus+, mCherry− (green, G1/G0) endothelial cells were exclusively EdU−, showing that the Cdt1-mVenus reporter does not label S phase cells in vivo (Fig. 2D–D**’** Fig. 2E top row, Fig. 2F, Supp. Fig. 2A). In contrast, mVenus−, mCherry + cells (red, S) dominated (97.9%) PIP-FUCCI-labeled EdU-labeled cells (Fig. 2D–D**’**, Fig. 2E second row; Fig. 2F), showing that the Gem-mCherry reporter faithfully labeled endothelial cells in S phase in vivo. While most (89%) of mCherry+ endothelial cells are EdU+, some mCherry+ cells score as EdU− (Supp. Fig. 2A), perhaps reflecting that detection of EdU labeling in tissues is likely not as sensitive as the expressed reporter. mVenus+, mCherry+ endothelial cells (orange, G2) were primarily EdU-, although a small proportion (5%) were EdU+ (Fig. 2D**’**, Fig. 2E bottom row, Fig. 2F, Supp. Fig. 2A). Since EdU labeling occurs over 2 h, some endothelial cells were likely labeled in S but transitioned to G2 prior to harvest.

Ki67 reactivity identifies cells in late G1, S, and G2, although expression is heterogeneous rather than uniformly negative in G0 and G1 [33]. To further validate PIP-FUCCI readouts, we examined the relationship of the PIP-FUCCI reporter with Ki67 (Fig. 2G–I; Supp. Fig. 2B). Among PIP-FUCCI labeled retinal endothelial cells, mVenus−, mCherry+ (red, S) and mVenus+, mCherry+ (orange, G2) endothelial cells were almost exclusively Ki67+, consistent with our expectation that these cells were in the cell cycle in vivo (Fig. 2G’; Fig. 2H second and bottom row; Fig. 2I, Supp. Fig. 2B). In contrast, PIP-FUCCI-labeled Ki67− cells were almost exclusively mVenus + , mCherry− (green, 99.2%) (Fig. 2G’; Fig. 2H top row green arrowheads; Fig. 2I), consistent with our prediction that mVenus labels G1/G0 endothelial cells. While a majority (76%) of mVenus+, mCherry− cells were Ki67− (Supp. Fig. 2B), indicating they are likely in G0 or early G1 in vivo, some (24%) mVenus + , mCherry- (green) endothelial cells were Ki67+ (Fig. 2G’; Fig. 2H top row, white arrowhead; Supp. Fig. 2B), likely due to the accumulation of Ki67 in late G1 and/or its perdurance in early G0.

**Fig. 3:**
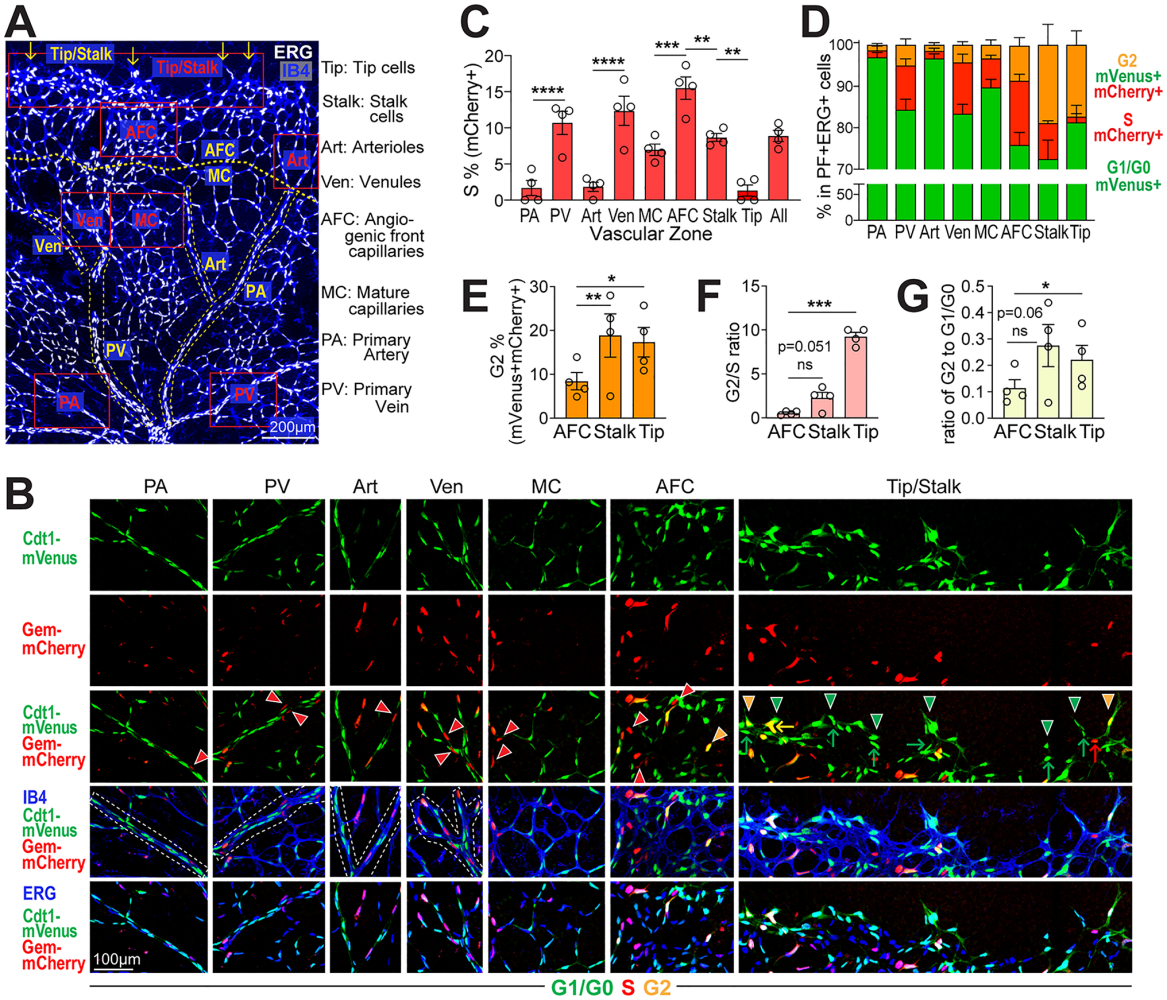
Spatial analysis reveals cell cycle differences in different retinal vascular zones. **A** A representative ERG (white)/IB4 (blue) labeled P6 retina image. Yellow dotted lines and labels, vascular zones used for pipeline quantification of endothelial cells cycle status; yellow arrows, endothelial cells defined as tip/stalk cells; red boxes, areas shown with additional markers and resolution in panel B. Label definitions to right. Scale bar, 200 μm. **B** High resolution views with additional markers of areas in (**A**) denoted by red boxes. ERG + endothelial cells labeled mVenus+, mCherry− (G1/G0), green arrowheads/arrows; mVenus-, mCherry+ (S), red arrowheads/arrows; and mVenus+, mCherry+ (G2M), yellow arrowheads/arrows; in labelled retinal vascular zones. In Tip/Stalk panel: arrows, stalk cells; arrowheads, tip cells. White dotted lines, PA, PV, Art and Ven outlines. Scale bar, 100 μm. **C** Quantification of % mVenus-, mCherry+ (S phase), ERG+ endothelial cells across vascular zones. All reporter-labelled ERG+ cells from each retina were quantified. ****p < 0.0001, ***p < 0.001, **p < 0.01 by one-way ANOVA & Sidak’s multiple comparisons test. **D** Quantification of endothelial cells (ERG+) labeled G1/G0, S and G2M in retinal vascular zones. All reporter-labelled ERG+ cells from each retina were quantified. **E–G** Comparison of AFC, Stalk and Tip cells.** E** Quantification of % mVenus+, mCherry+, ERG+ (G2) endothelial cells. **p < 0.01, *p < 0.05 by one-way ANOVA & Sidak’s multiple comparisons test. **F** Ratio of endothelial cells in G2 to S, **G** ratio of endothelial cells in G2 to G1/G0 in AFC vs. tip cells vs. stalk cells. *p < 0.05, ***p < 0.001 by paired t test. n = 4 pups for all quantifications

Closer inspection of retinal images revealed that endothelial cells in the angiogenic front exhibited signal consistent with G1/G0 (green), S (red), or G2 (orange) cell cycle stages, while cells in the mature region were largely G1/G0 (green) (Fig. 2B; Supp. Fig. 2C–C’), suggesting spatial differences in the distribution of cell cycle stages. One advantage to postnatal retinal angiogenesis analysis is that a temporal gradient of remodeling (optic nerve outward) vs. angiogenic expansion (distal to remodeling) exists, with a clear definition of tip cells at the front vs. stalk cells behind the tip vs. non-tip/non-stalk angiogenic front cells right behind the tip/stalk area [4]. Spatial domains for large arteries, arterioles, and large veins and venules that form upon remodeling are also well-defined. To better understand how the cell cycle changes with time and vascular maturation, we developed a novel semi-automated zonation pipeline that identified the proportion of endothelial cells in G1/G0 vs. S vs. G2 in primary arteries (PA), Arterioles (Art), primary veins (PV), venules (Ven), mature capillaries (MC), stalk cells (Stalk), tip cells (Tip) and angiogenic front capillaries (AFC) (Fig. 3A (yellow dotted lines, zones used in pipeline; red boxes, regions shown at higher resolution in panel B); Supp. Fig. 2D, customized scripts on GitHub, see Methods).

As expected, the proportion of mVenus-, mCherry + (S phase) endothelial cells was elevated in primary veins and venules compared to arterial counterparts (Fig. 3B third row, red arrowheads in PA, PV, Art, Ven; Fig. 3C), consistent with reports that veins are more proliferative [2, 9]. Interestingly, primary arteries and arterioles consistently have a small proportion (~ 2%) of S-phase labeled endothelial cells (Fig. 3C), suggesting that a small proportion of arterial endothelial cells are in the cell cycle and not quiescent, consistent with a recent report showing that 2.5% arterial endothelial cells are mitotic in neonatal coronary arteries [34]. The highest S phase labeling was seen in the AFC (angiogenic front capillaries, 15.5%), which is significantly higher than the more mature MC zone (mature capillaries, 7%, Fig. 3C; Fig. 3B third row, red arrowheads in MC, AFC) and stalk cells (8.7%, Fig. 3C; Fig. 3B third row, red arrow in Tip/Stalk). Very few tip cells are mVenus−, mCherry+, (S phase, 1%, Fig. 3C; Fig. 3B third row Tip/Stalk), consistent with a previous report that tip cells do not usually divide [15].

Further analysis of the high-resolution zonation of cell cycle status in retinal endothelial cells revealed a surprising increase in the proportion of mVenus+, mCherry+ endothelial tip cells (orange, G2, 17.3%) and stalk cells (18.9%) (Fig. 3D–E) compared to neighboring endothelial cells in the angiogenic front (AFC, 8.4%) (Fig. 3E; Fig. 3B third row, yellow arrowheads and arrows in AFC and Tip/Stalk). Additionally, while other vascular zones had 1–3 times more endothelial cells in S than G2 phase (Fig. 3D), tip cells showed a significantly higher G2/S ratio compared to the angiogenic front capillaries (AFC) just behind the tip/stalk (9.3 in tip cells vs. 0.6 in AFC), while stalk cells showed a non-significant increase in this ratio (2.3) over AFC due to increased S-phase cells (Fig. 3F). This trend is also seen for both tip and stalk cell categories in the G2/G1 ratio comparison that is significantly higher in tip cells compared to AFC (0.22 in tip cells vs. 0.11 in AFC, Fig. 3G) and trending for stalk cells (0.28) vs. AFC, consistent with the idea that G2-enrichment of tip cells is not simply due to decreased S phase tip cells. Thus, a portion of endothelial tip and stalk cells may be held in a G2 arrest, which suggests co-ordination of the cell cycle and cell behaviors such as sprout extension.

## Conclusions

This study validates the use of a novel PIP-FUCCI cell cycle reporter in primary endothelial cells and in vivo. This reporter allows for precise determination of both G1/S and S/G2 transitions not found in earlier versions, resulting from the use of the PIP degron of Cdt1_1-17_ instead of Cdt1_30-120_ in previous reporters [24]. As expected, live image analysis of primary endothelial cells revealed an average cell cycle transit time of about 16 h, with cells transiting G1/G0, S, and G2 prior to mitosis. This precise cellular readout of the cell cycle will allow for a better understanding of how endothelial cell cycle status influences its responses, as was recently shown for vascular endothelial cells in early vs. late G1 [11], and cell behaviors such as migration.

In vivo analysis of the PIP-FUCCI reporter took advantage of the stereotypical expansion of the postnatal mouse retinal vasculature, and we validated that the reporter readouts coincided with more traditional measures of cell cycle in vivo. Analysis using a novel semi-automated pipeline revealed that endothelial cells in the vascular front and veins had higher percentages of S-phase cells than arteries, and that tip cells were predominantly in either G1/G0 or G2, as predicted from their non-proliferation profile [35]. The dearth of S phase endothelial tip cells likely results from elevated levels of VEGF-A signaling [15] and is predicted to allow for migration over proliferation. Non-proliferative migration is a feature of tip cells in many but not all vascular beds; for example, many tip cells in zebrafish intersegmental vessels sprout in S/G2/M and divide as they migrate from the dorsal aorta [18].

The enhanced cell cycle stage delineation of the PIP-FUCCI reporter unexpectedly led to the discovery of significant numbers of tip and stalk cells in the G2 phase of the cell cycle. This is the first reporter delineation of G2 in retinal angiogenesis, since G2-specific antibodies are rare and previous reporters did not precisely define S/G2 [19, 36]**.** Moreover, the ratio of G2 tip cells to tip cells in other cell cycle phases (S and G1/G0) was significantly skewed relative to nearby stalk cells that had elevated G2 proportions but also significant S-phase cells, and angiogenic front cells behind the tip whose G2/S ratio was more canonical. These relationships indicate that endothelial cells are stalled or arrested in G2 in the tip cell and stalk cell domains, along with numerous cells that are in G1/G0. The concept of a G2 stall has been described in other developmental models, such as *Drosophila* eye and sensory organ development [37, 38], as a means of co-ordinating morphogenetic movements and developmental programs. Endothelial cells in the stalk compete for the tip cell position and change positions over time [39, 40], but endothelial cells in the tip cell position rarely if ever undergo mitosis [15]. Thus, it is conceivable that an endothelial cell becomes permissive to adopt a tip cell phenotype at the S/G2 transition, and if it then moves into the tip position, a normally short G2 is extended (or stalled) to prevent mitosis during the time it resides at the tip (about 4–8 h) [39, 40]. A similar mechanism may also enrich for G2 in stalk cells that may require intermediate proliferative capacity to match with tip cell dynamics. We anticipate that future studies using this new tool will better define the integration of the vascular cell cycle in various tissues during development and disease.

## Supplementary Information

Below is the link to the electronic supplementary material.

Supplementary file1 (PDF 1903 KB)

Supplementary file2 (AVI 1069 KB) Supplementary Movie 1 (link to Figure 1B). Time-lapse imaging of representative PIP-FUCCI-expressing HUVEC. One endothelial cell cycle with mVenus+, mCherry- (G1, green), mVenus-, mCherry+ (S, red), and mVenus+, mCherry+ (G2, orange) phases. Frames start after cytokinesis through next mitosis. Image acquisition, 1 frame/10min. Scale bar, 50 μm.

Supplementary file3 (AVI 13571 KB) Supplementary Movie 2 (linked to Supp. Figure 1A). Time-lapse imaging of representative PIP-FUCCI-expressing HUVEC in G1 phase. Green line, cell migration trace. Image acquisition, 1 frame/10min. Scale bar, 20μm.

Supplementary file4 (AVI 5327 KB) Supplementary Movie 3 (linked to Supp. Figure 1A). Time-lapse imaging of representative PIP-FUCCI-expressing HUVEC in S phase. Green line, cell migration trace. Image acquisition, 1 frame/10min. Scale bar, 20μm.

Supplementary file5 (AVI 1111 KB) Supplementary Movie 4 (linked to Supp. Figure 1A). Time-lapse imaging of representative PIP-FUCCI-expressing HUVEC in G2 phase. Green line, cell migration trace. Image acquisition, 1 frame/10min. Scale bar, 20μm.
