# Supplementary material for: Differential endothelial cell cycle status in postnatal retinal vessels revealed using a novel PIP-FUCCI reporter and zonation analysis
Source: Angiogenesis. 2024 May 25;27(4):681–9. doi: 10.1007/s10456-024-09920-0 (PMC11564245; doi:10.1007/s10456-024-09920-0)
Supplement: Supplementary file 1 — Supplementary file1 (PDF 1903 KB) [file 10456_2024_9920_MOESM1_ESM.pdf]

Differential endothelial cell cycle status in postnatal retinal vessels revealed using a novel PIP-FUCCI reporter and zonation analysis

Ziqing Liu[1#], Natalie T Tanke[2], Alexandra Neal[1], Tianji Yu[1], Tershona Branch[1], Arya Sharma[1], Jean G Cook[3], Victoria L Bautch[1,2,4,5*]

[1]Department of Biology, [2]Curriculum in Cell Biology and Physiology, [3]Department of Biochemistry and Biophysics, [4]McAllister Heart Institute, [5]Lineberger Comprehensive Cancer Center, The University of North Carolina, Chapel Hill, NC USA

* corresponding author

[#] present address: Dept of Physiology, Medical College of Wisconsin, Milwaukee WI

SUPPLEMENTAL FILE:

1. Supplemental Figures (2)
2. Supplemental Resource Tables (2)

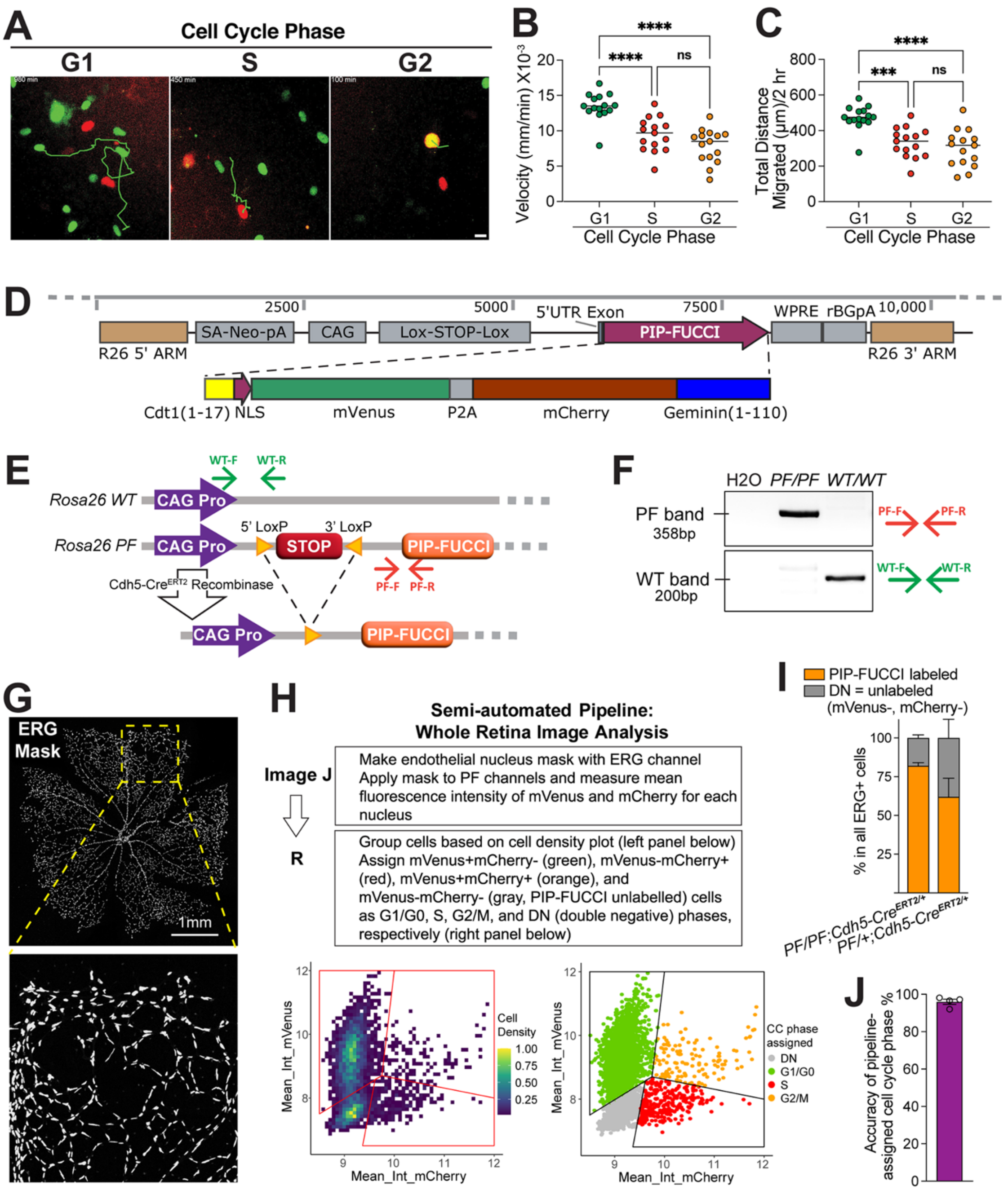

Supplementary Figure 1. PIP-FUCCI reporter cells and mice and workflow for retina analysis. (A) Representative cell migration traces (green lines) for indicated cell cycle phases. Scale bar, 50µm. **(B)** Quantification of velocity per 2 hr segment for indicated cell cycle phases. (n=15 cells/phase, 3 replicate movies). **(C)** Quantification of total distance migrated per 2 hr segment for indicated cell cycle phases (n=15 cells/phase, 3 replicate movies). **** p< 0.0001, *** p< 0.001, by one-way ANOVA & Tukey's multiple comparisons test. **(D)** Construct used to generate the PIP-FUCCI reporter mouse line. **(E)** Location of genotyping primers. **(F)** Example of genotyping results on DNA agarose gel. **(G)** Example of ERG mask. **(H)** Workflow for whole retina

analysis with ERG mask. **(I)** Quantification of % PIP-FUCCI labeled cells in ERG+ endothelial cells from *PF/PF*;*Cdh5-Cre*$^{ERT2/+}$ or *PF/+*;*Cdh5-Cre*$^{ERT2/+}$ retinas (n= 2 pups for each genotype). **(J)** Accuracy of manual vs. algorithm-assigned cell cycle phases from the same subset of endothelial cells of the same retinal images (PIP-FUCCI labeled and stained for IB4 and ERG). n = 4 pups.

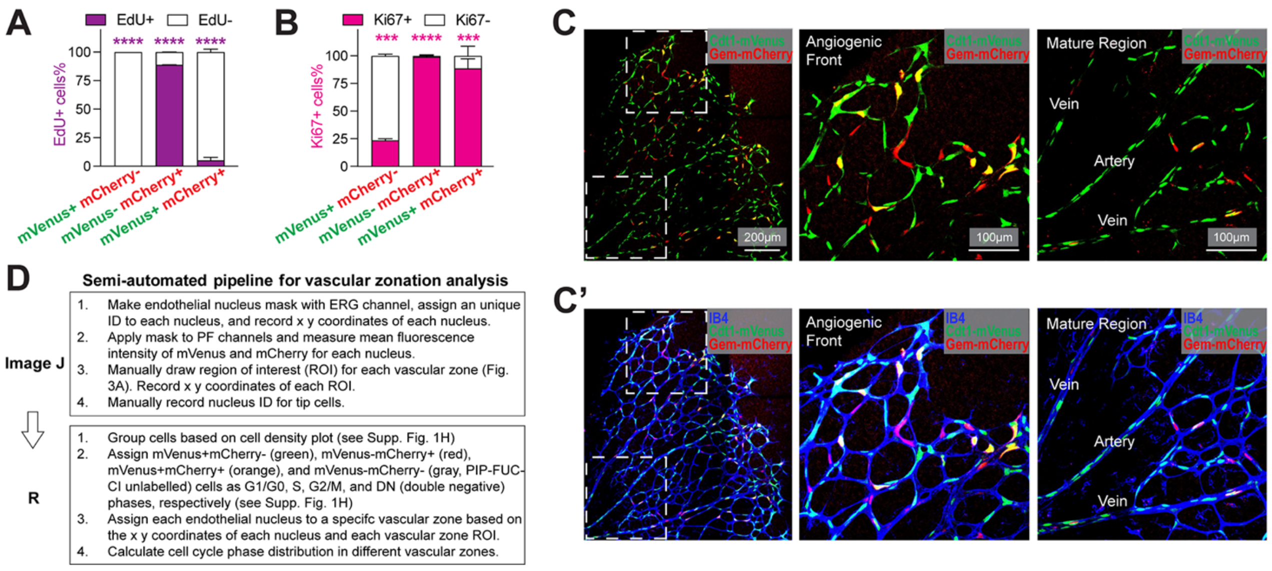

Supplementary Figure 2. Workflow for vascular zonation analysis of the retina.

(A) Quantification of retinal endothelial cells EdU labeled relative to PIP-FUCCI status (related to **Fig. 2D-F)**. n = 3 pups. **** p< 0.0001 by two-way ANOVA & Sidak's multiple comparisons test. **(B)** Quantification of Ki67+ retinal endothelial cells relative to PIP-FUCCI status (related to **Fig. 2G-I)**. n = 2 pups. **** p< 0.0001, *** p<0.001 by two-way ANOVA & Sidak's multiple comparisons test. **(C-C')** Representative image of one leaflet of a *PF/PF*;*Cdh5-Cre*$^{ERT2/+}$ retina stained for IB4 and ERG (same imaging fields as **Fig. 2B**, IB4 pseudo-colored blue in C'). Boxed areas in far left panel (scale bar, 200 μm) magnified (scale bar, 100 μm) in middle (Angiogenic Front) and far right (Mature Region) panels. **(D)** Workflow for semi-automated vascular zonation analysis of PIP-FUCCI retinal images with ERG mask.

SUPPLEMENTARY TABLES

Supplementary Table 1. PCR Primers

Primer Name	Primer Sequence	Expected Products
Rosa26 WT-F<br>Rosa26 WT-R	TGGAGTTGCAGATCACGAGG<br>TGTTTTGGAGGCAGGAAGCA	WT = 225 bp<br>PF = no band
Rosa26 PF-F<br>Rosa26 PF-R	GCTAACCATGTTCATGCCTTC<br>CGCCCTCGCCGGACACGCTGAAC	WT = no band<br>PF = 357 bp
GenCre F<br>GenCre R	GACCAGGTTCGTTCACTCA<br>TAGCGCCGTAAATCAAT	WT = no band<br>Cre = 400 bp

Supplementary Table 2. Antibodies

Antibody	Species	Company	Catalog No.	Dilution	Final Conc
IsolectinB4-biotin	NA	ThermoFisher	I21414	1:100	10 µg/ml
Streptavidin-Alexa405	NA	ThermoFisher	S32351	1:50	20 µg/ml
Ki67-Alexa647	Rat	Biolegend	652407	1:20	25 µg/ml
ERG-Alexa647	Rabbit	Abcam	ab196149	1:100	5 µg/ml
DAPI	NA	Sigma	10236276001	1:1,000	10 ng/ml
